# Parkin Regulation and Neurodegenerative Disorders

**DOI:** 10.3389/fnagi.2015.00248

**Published:** 2016-01-12

**Authors:** Cheng-Wu Zhang, Liting Hang, Tso-Pang Yao, Kah-Leong Lim

**Affiliations:** ^1^Neurodegeneration Research Laboratory, National Neuroscience InstituteSingapore, Singapore; ^2^Institute of Advanced Materials, Nanjing Tech UniversityNanjing, People’s Republic of China; ^3^Department of Physiology, National University of SingaporeSingapore, Singapore; ^4^Departments of Pharmacology and Cancer Biology, Duke University Medical CenterDurham, NC, USA; ^5^Duke-NUS Graduate Medical School, National University of SingaporeSingapore, Singapore

**Keywords:** Parkinson’s disease, mitophagy, proteasome, ubiquitin, neurodegeneration, autophagy, protein misfolding, mitochondria

## Abstract

Parkin is a unique, multifunctional ubiquitin ligase whose various roles in the cell, particularly in neurons, are widely thought to be protective. The pivotal role that Parkin plays in maintaining neuronal survival is underscored by our current recognition that Parkin dysfunction represents not only a predominant cause of familial parkinsonism but also a formal risk factor for the more common, sporadic form of Parkinson’s disease (PD). Accordingly, keen research on Parkin over the past decade has led to an explosion of knowledge regarding its physiological roles and its relevance to PD. However, our understanding of Parkin is far from being complete. Indeed, surprises emerge from time to time that compel us to constantly update the paradigm of Parkin function. For example, we now know that Parkin’s function is not confined to mere housekeeping protein quality control (QC) roles but also includes mitochondrial homeostasis and stress-related signaling. Furthermore, emerging evidence also suggest a role for Parkin in several other major neurodegenerative diseases including Alzheimer’s disease (AD) and Amyotrophic Lateral Sclerosis (ALS). Yet, it remains truly amazing to note that a single enzyme could serve such multitude of functions and cellular roles. Clearly, its activity has to be tightly regulated. In this review, we shall discuss this and how dysregulated Parkin function may precipitate neuronal demise in various neurodegenerative disorders.

## Introduction

Parkin is a unique multifunctional ubiquitin ligase whose various roles in the cell, particularly in neurons, are widely thought to be protective. The pivotal role that Parkin plays in maintaining neuronal survival is underscored by our current recognition that Parkin dysfunction represents not only a predominant cause of familial Parkinsonism but also a formal risk factor for the more common, sporadic form of Parkinson’s disease (PD; Dawson and Dawson, 2014). Moreover, emerging evidence also implicates a role for Parkin in Alzheimer’s disease (AD), Amyotrophic Lateral Sclerosis (ALS) and Huntington’s disease (HD; Tsai et al., 2003; Rosen et al., 2010; Hebron et al., 2014). Further, several studies have documented the ability of Parkin to protect neurons against a wide variety of insults, including those mediated by neurotoxins and metallic ions (Kubo et al., 2013). Thus, maintaining an optimal state of Parkin function appears to be an important determinant of neuronal health in general, although dopaminergic (DA) neurons whose degeneration is causative of PD seems particularly sensitive to Parkin deficiency.

Mutations in Parkin were originally identified in Japan more than a decade ago to be a major genetic contributor of Autosomal Recessive Juvenile Parkinsonism (ARJP; Kitada et al., 1998). Following this discovery, several ethnically diverse individuals with early-onset PD (age <45 years) in other parts of the world were also found to carry Parkin mutations, which occur at a frequency of about 10–20 and 50% in sporadic and familial early-onset cases respectively (Lücking et al., 2000; Periquet et al., 2003; Mata et al., 2004). At around the same period, three independent groups demonstrated that Parkin functions as an E2-dependent ubiquitin ligase associated with the ubiquitin-proteasome system (UPS; Imai et al., 2000; Shimura et al., 2000; Zhang et al., 2000), a major intracellular protein degradation machinery, which fuelled intense research on the role of Parkin in neuronal protein homeostasis. The particular excitement in this topic was in part also due to the finding that the first PD-linked gene elucidated before Parkin, i.e., α-synuclein, encodes a presynaptic protein that tends to misfold and that was subsequently found to be a major component of Lewy Bodies (LBs), a histological hallmark of the PD brain (Spillantini et al., 1997). It seemed intuitive therefore to associate UPS dysfunction with PD pathogenesis. Further support for this soon came with the elucidation of another PD-linked gene, i.e., UCH-L1, which encodes yet another component of the UPS. One could therefore readily appreciate that it was then a hugely popular trend to look at the “proteasome theory of PD” (and subsequently, the related “autophagy theory of PD”). Parkin was soon found to be involved in both UPS- and autophagy-associated protein quality control (QC; Lim and Zhang, 2013). However, along the way, several groups (particularly those working with *Drosophila* Parkin models) also documented an intriguing relationship between Parkin and mitochondrial QC, i.e., mitochondria tends to become abnormal in the absence of functional Parkin (Greene et al., 2003). This phenomenon remains largely unexplained and overlooked until 2008, when a seminal discovery by a team led by Richard Youle demonstrated that Parkin is a key mammalian regulator of mitochondrial autophagy, or “mitophagy” (Narendra et al., 2008). Subsequent studies by his group and several others revealed that Parkin collaborates with another PD-linked gene product known as PINK1 (encoding a mitochondrial-targeted serine/threonine kinase) to mediate mitophagy (Geisler et al., 2010; Matsuda et al., 2010; Narendra et al., 2010a; Vives-Bauza et al., 2010). Collectively, these reports triggered an explosion of interest among the global mitochondria and PD research community in delineating the pathways involved in Parkin/PINK1-mediated mitophagy, with the excitement ensuing to this date. Meanwhile, the “mitochondrial theory of PD” that was popular in the eighties and nineties but had lay “dormant” subsequently is now enjoying its “renaissance” of support.

Notwithstanding the periodic bias in favor of a particular disease-associated pathway, it is now evident that Parkin is an important regulator of protein and mitochondrial homeostasis that operates against the backdrop of a multitude of intracellular pathways. Accordingly, the ubiquitin ligase itself would need to be exquisitely regulated to fulfil its diverse cellular roles in a timely fashion to maintain optimal neuronal function. In this review, we shall discuss the mechanisms underlying Parkin activity regulation and its involvement in protein/mitochondrial homeostasis and finally, how dysregulated Parkin function may predispose neurons to degeneration.

## Structure and Regulation of Parkin Activity

**Figure 1:**
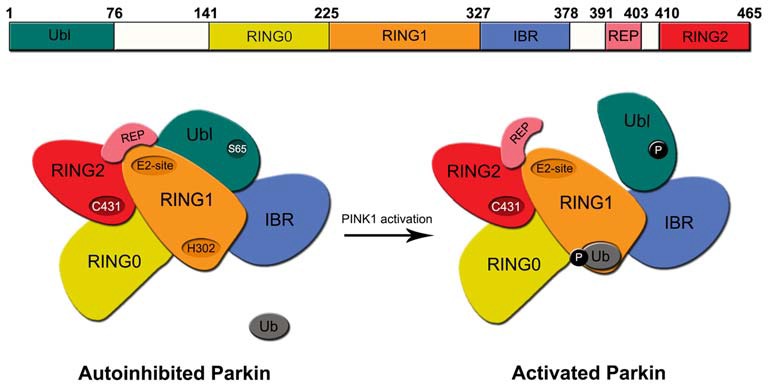
**Structure of regulation of Parkin.** *Top*, Schematic depiction of parkin structure. *Bottom*, A model of Parkin activity regulation—Under normal conditions, Parkin exists in an auto-inhibited state where access to its E2-binding RING1 site is occluded by its Ubl and repressor element of parkin (REP) domains and access to its RING2’s active site is blocked by the RING0 domain. Upon the phosphorylation Parkin’s Ubl domain by PINK1 at Serine 65 (S65) and the concomitant engagement of RING1 with phosphorylated ubiquitin, the Ubl is displaced away from RING1, which led to the structural rearrangement of the various domains of Parkin. The enzyme consequently becomes fully activated.

Structurally, the 465 amino acid-containing human Parkin protein is comprised of a ubiquitin-like (Ubl) domain at its N-terminus, a RING1-IBR-RING2 (RBR) domain at its C-terminus and a unique middle segment that links the two domains. A zinc-chelating RING0 domain that is juxtaposed (N-terminally) to the RBR domains resides within the linker segment. Another motif, known as the Repressor Element of Parkin (REP), sits between the IBR and RING2 domains (Figure 1). During Parkin-mediated ubiquitination, E2 enzymes are first recruited to RING1 domain and the charged ubiquitin they carry are then transferred to a catalytic cysteine (C431) in the RING2 domain before being finally transferred to the primary amino group of the substrate via an iso-peptide bond formation. This is an intriguing catalytic mechanism for a RING-containing E3 as the process is reminiscent of HECT domain-containing ubiquitin ligases. We now know that RBR E3 s such as Parkin and HHARI use such a RING-HECT hybrid mechanism for catalysis (Wenzel et al., 2011). Interestingly, structural studies of Parkin revealed that the enzyme exists in a closed, inactivated state under normal conditions. This auto-inhibited state is achieved through an intricate folding of the protein whereby RING0 is inserted between RING1 and RING2 and in so doing occludes the active site on RING2. At the same time, the closed conformation also promotes the REP to adopt a structure that lies across the putative E2-binding site on RING1 thus preventing its recruitment of E2 s (Riley et al., 2013; Trempe et al., 2013; Wauer and Komander, 2013; Figure 1). Consistent with this, both Helen Walden’s group and ours have shown experimentally that the N-terminal region of Parkin represses Parkin-mediated polyubiquitination, although an important difference between the two studies is the repressing element involved, i.e., we have proposed the linker segment (amino acid 152–237 – that contains the RING0 domain) to be the repressing element whereas Walden’s group showed that it is the Ubl domain (Chaugule et al., 2011; Chew et al., 2011). Notwithstanding this, for Parkin to become active, it is obvious that substantial rearrangements of its structure need to take place to allow the dissociation of RING0 from RING2 as well as REP from RING1 among other conformational changes.

Recent studies have revealed that Parkin activity may be stimulated by a two-step process that involves the phosphorylation of serine 65 (S65) in its Ubl domain (Matsuda et al., 2010; Kondapalli et al., 2012; Shiba-Fukushima et al., 2012) and the phosphorylation of ubiquitin at the same site (Kane et al., 2014; Koyano et al., 2014). Interestingly, whereas phosphorylation of Parkin’s Ubl domain promotes its dissociation from RING1, the phosphorylation of ubiquitin (also at S65) enhances its association with RING1. Thus, despite the fact that Ubl and ubiquitin share marked sequence similarities, their respective interaction with RING1 of Parkin is oppositely regulated by phosphorylation. Precisely how these phosphorylation events could alter the multiple intra-molecular interactions that define the auto-inhibited enzyme to bring about its activation was unclear until very recently. A number of new structural studies on Parkin have now clarified the mechanism of Parkin activation (Kumar et al., 2015; Sauvé et al., 2015; Wauer et al., 2015). In essence, the concerted finding is that the dissociation of Ubl from RING1 (following S65 phosphorylation) exposes a histidine residue (H302) on RING1 that binds to phosphorylated ubiquitin, which leads to the displacement the Ubl domain from its original conformation, loss of structure near the RING1/IBR interface and the overall relieve of Parkin’s auto-inhibited state (Figure 1). RING1 now becomes available for the engagement with E2~ubiquitin conjugate to trigger downstream substrate ubiquitination. Although these structural studies have certainly contributed to a clearer picture of Parkin activation, how the activated enzyme returns to its basal auto-inhibited state remains elusive. Presumably, this will involve the dephosphorylation of S65-phosphorylated Ubl domain and ubiquitin by an unknown phosphatase(s). Moreover, because the above model of Parkin activation is related to its function in mitophagy, it is also unclear whether the same activation mechanism is utilized to promote Parkin-mediated ubiquitination in non-mitophagy events. Parkin’s role in the cell is certainly not restricted to protein turnover during mitophagy. As a matter of fact, the majority of reports before 2008 described its role in protein QC that is not directly related to mitophagy.

## Parkin and Protein Homeostasis

As mentioned earlier, Parkin functions as an E3 ligase associated with the UPS, a major proteolytic machinery that normally identifies and degrades unwanted intracellular proteins. In this system, proteins that are destined for proteasome-mediated degradation are added a chain of ubiquitin via a reaction cascade that involves the ubiquitin-activating (E1), -conjugating (E2) and -ligating (E3) enzymes. Through the sequential and repetitive actions of these enzymes, successive isopeptide linkages are formed between the C-terminal glycine carboxyl group (G76) of the ubiquitin moiety being added and the α-amino group of a free lysine (most commonly K48) on the ubiquitin that is attached to the protein. The (G76-K48) polyubiquitinated substrate is then recognized by the 26S proteasome as a target for degradation. It is important to highlight that the ubiquitin sequence contains seven lysine residues (at positions 6, 11, 27, 29, 33, 48 and 63) and that polyubiquitin chain assembly can occur at any of these lysine residues (Peng et al., 2003). In addition, proteins can also be monoubiquitinated. Notably, both K63-linked polyubiquitination and monoubiquitination of proteins are not typically associated with proteasome-mediated ubiquitination.

Along with the original discovery by three independent groups who demonstrated that Parkin functions as a ubiquitin ligase, they also found that disease-associated Parkin mutations compromise its role as an E3 enzyme (Imai et al., 2000; Shimura et al., 2000; Zhang et al., 2000). A logical and popular hypothesis that ensued is that loss of Parkin function would lead to the toxic accumulation of one or several of its substrates, thereby leading to neurodegeneration. This had fuelled intense effort by many laboratories around the world to identify the culprit substrate(s) involved. To date, no less than 25 substrates (or putative substrates) of Parkin have been reported (Table 1). However, none of the Parkin substrates identified thus far is exclusively expressed in DA neurons, which raises the question on why DA neurons in familial Parkinsonism cases linked to Parkin mutations are rather selectively vulnerable to deficient Parkin function. Of course, one could argue that neither is Parkin expression confined to DA neurons and therefore it is perhaps not surprising to note the broad distribution of its substrates as well. Importantly, few from the laundry list of Parkin substrates fulfil an important criterion expected of an “authentic” Parkin substrate, i.e., accumulation in the brains of ARJP patients and Parkin-deficient models. Although the pace of substrate identification has slowed down considerably in recent years, new Parkin substrates continue to emerge periodically, especially those related to the mitochondria. Among the recently isolated Parkin substrates is the zinc finger-containing protein called PARIS (ZNF746; Shin et al., 2011), a major transcriptional repressor of PGC-1α expression, which in turn regulates the transcription of many genes involved in cellular metabolism. Importantly, unlike most previously identified Parkin substrates, PARIS was shown to accumulate in post-mortem brain tissues derived from ARJP and sporadic PD patients, as well as in the ventral midbrain region of mice that is conditionally ablated of Parkin expression (Shin et al., 2011). Based on these findings, the authors proposed that PARIS is an “authentic” Parkin substrate. More recently, and some may think curiously, Parkin was found to interact with the anaphase promoting complex/cyclosome (APC/C) coactivators Cdc20 and Cdh1 to mediate the degradation of several key mitotic regulators independent of APC/C. Accordingly, its deficiency results in the elevated expression of these substrates leading to mitotic defects, genomic instability and tumorigenesis (Lee et al., 2015). Corroborating with these findings, we and others have previously similarly shown that Parkin dysfunction underlies several types of cancers (Cesari et al., 2003; Tay et al., 2010; Yeo et al., 2012). Although not directly related to the current review topic, the several recent reports collectively pointing towards a role for Parkin as a tumor suppressor suggest the interesting possibility that Parkin-related neurodegeneration may arise from aberrant cell cycle re-entry in post-mitotic neurons.

**Table 1:** **A selection of reported Parkin substrates**.

Substrates	Ub Type	Elevated			Reference
		KO mice	ARJP brain	PD brain
Ataxin-2	–	Yes	–	–	Huynh et al. (2007)
Ataxin3 polyQ79	–	–	–	–	Tsai et al. (2003)
Bcl-2	Mono	–	–	–	Chen et al. (2010)
CDCrel-1	–	Yes/No	Yes/No	–	Ko et al. (2005)
CDCrel-2a	–	–	Yes	–	Choi et al. (2003)
Cyclin E	–	No	Yes/No	Yes	Staropoli et al. (2003) and Ko et al. (2005)
DJ-1 L166P	K63	–	–	–	Olzmann et al. (2007)
Dopamine Transporter	–	–	–	–	Jiang et al. (2004)
Drp1	K48	–	–	–	Wang et al. (2011a)
ps15	Mono	–	–	–	Fallon et al. (2006)
FBP1	–	Yes	Yes	Yes	Ko et al. (2006)
Fbw7β	K48	Yes	Yes	–	Ekholm-Reed et al. (2013)
Hsp70	Multiple	No	No	Yes	Moore et al. (2008)
	Mono
LIM Kinase	–	–	–	–	Lim et al. (2007)
Mitofusin	–	–	–	–	Poole et al. (2010) and Ziviani et al. (2010)
*O*-glycosylated α-synuclein	–	–	Yes	–	Shimura et al. (2001)
P38/AIMP2	Multiple	Yes	Yes	Yes	Corti et al. (2003); Ko et al. (2005); Periquet et al. (2005) and Hampe et al. (2006)
	Mono
Pael-R	–	No	Yes/No	–	Ko et al. (2005)
PARIS (ZNF746)	K48	Yes	Yes	Yes	Shin et al. (2011)
PDCP2-1	–	–	Yes	Yes	Fukae et al. (2009)
Phospholipase C**γ**1	–	Yes	–	–	Dehvari et al. (2009)
PICK1	Mono	No	–	–	Joch et al. (2007)
RanB2	–	–	–	–	Um et al. (2006)
Synaptotagmin XI	–	Yes/No	–	–	Periquet et al. (2005)
Synphilin-1	K63	No	No	–	Chung et al. (2001); Ko et al. (2005) and Lim et al. (2005)
VDAC1	K27, Mono	Yes	–	–	Periquet et al. (2005); Geisler et al. (2010) and Narendra et al. (2010b)
α/β tubulin	–	Yes/No	No	–	Ren et al. (2003) and Ko et al. (2005)

**Figure 2:**
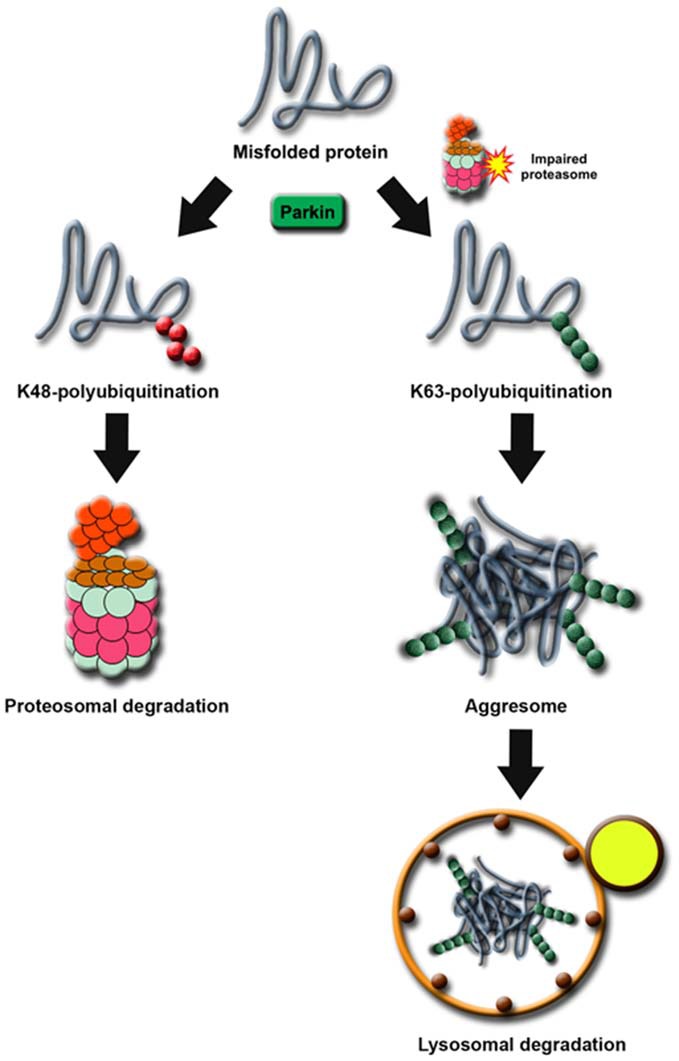
**Proposed model of Parkin’s role as a triage between proteasome and autophagy degradation.** Under normal cellular conditions, proteins destined for degradation by the proteasome are tagged with a chain of K48-linked ubiquitin. In times of proteolytic stress, the cell switches to K63-linked ubiquitination to divert the protein load originally targeted for proteasomal degradation away from the otherwise overloaded machinery. Parkin facilitates this switch by increasing its affinity for Ubc13 in the presence of proteasome dysfunction.

Notwithstanding that only a handful of substrates reported to date could fulfil the restricted criterion of a Parkin substrate (as mentioned above), what constitutes an “authentic” substrate for Parkin is really debatable. Although protein ubiquitination is classically associated with proteasome-mediated degradation, the existence of non-classic ubiquitin modifications such as K63-linked polyubiquitination would caution against the fixation on the traditional view that substrates of a ubiquitin ligase must exhibit an accelerated, proteasome-dependent turnover in the presence of the enzyme. This is particularly relevant to Parkin, which we and others have demonstrated to be a multifunctional enzyme capable of mediating alternative ubiquitin topologies such as monoubiquitination and K63-linked polyubiquitination—modifications that are typically uncoupled from the proteasome and often considered as “non-proteolytic” (Doss-Pepe et al., 2005; Lim et al., 2005; Hampe et al., 2006; Matsuda et al., 2006). Our results would suggest that the catalytic function of Parkin is not limited to targeting substrate for degradation by the proteasome. Thus, the lack of accumulation of an identified Parkin substrate in the brains of ARJP patients and Parkin-deficient models does not necessarily mean that it is less than an “authentic” substrate. For example, we have previously reported that Parkin-mediated polyubiquitination of synphilin-1 (an interactor of α-synuclein) normally occurs via K63-linked chains, which does not appear to affect its steady-state turnover (Lim et al., 2005). Not surprisingly, the level of synphilin-1 is neither appreciably altered in ARJP brains nor in brain tissues derived from Parkin null mice (Ko et al., 2005). A corollary to this is that proteasome-independent pathways may also be relevant to Parkin-related neurodegeneration. By virtue of its apparent dissociation from the proteasome, we have originally proposed that Parkin-mediated K63-linked ubiquitination may be involved in cargo diversion during proteasomal stress and accordingly, in the biogenesis of inclusion bodies associated with neurodegenerative diseases (Lim et al., 2006). Our proposal is consistent with the concept of “aggresomes”, which are juxtanuclear inclusion bodies formed in the presence of proteasomal stress and that have been suggested to act as staging grounds for the disposal of protein aggregates via the autophagic route (Kopito, 2000). Supporting our hypothesis, we found that Parkin-mediated K63 polyubiquitination of synphilin-1 promotes its aggregation into aggresome-like inclusion bodies (Lim et al., 2005). Corroborating our findings, Olzmann et al demonstrated that Parkin-mediated K63 polyubiquitination of misfolded DJ-1 couples the protein to the dynein motor complex via the histone deacetylase 6 (HDAC6) adaptor, thereby promoting its sequestration into aggresomes (Olzmann et al., 2007). Importantly, we further identified K63-linked polyubiquitin as a cargo selection signal for macroautophagy-mediated clearance of aggresomes (Tan et al., 2008a,b). Notably, Wang et al. (2012) demonstrated that the formation of aggresomes mediated by mutant superoxide dismutase 1 (SOD1) is dependent on ataxin 3-catalyzed editing of K63-linked polyubiquitin chain on SOD1 (presumably to a correct length) which supports our findings. In a similar development, Hao et al. (2013) demonstrated that the disposal of aggresomes requires the proteasomal deubiquitinating enzyme Poh1, which releases free (K63-linked) ubiquitin chains from aggresomes that activates HDAC6-dependent autophagy clearance of the cargo. In Poh1-deficient cells, aggresome clearance is blocked but may be rescued by the introduction of free K63-linked ubiquitin chains (via microinjection). Finally, by means of ubiquitin linkage-specific antibodies, we recently showed that proteasome inhibition indeed promotes K63-linked ubiquitination of proteins especially in Parkin-expressing cells (Lim et al., 2013). Importantly, we further demonstrated that the recruitment of Ubc13 (an E2 that mediates K63-linked polyubiquitin chain formation exclusively) by Parkin is selectively enhanced under conditions of proteasomal stress, which favors aggresome formation and thereby the clearance of Parkin substrates via autophagy (Lim et al., 2013). Thus, by being capable of mediating both proteasome-associated K48-polyubiquitination and macroautophagy-associated K63-linked polyubiquitination, Parkin may potentially act as an important triage between the two major cellular degradation systems (Figure 2). This multi-functionality of Parkin may in part help explain its apparent broad neuroprotective properties, as the flexibility of ubiquitin linkage usage presumably would allow the enzyme to adapt rapidly to changes in cellular environment.

## Parkin and Mitochondrial Homeostasis

One of the first hints that Parkin may play a role in mitochondrial homeostasis aside from its role as a regulator of protein turnover came from a study by Greene et al. (2003) in fruit flies. The authors analyzed adult *Drosophila parkin* null mutant and observed that the most prominent pathology is not in the brain but in the flight musculature of these mutant flies, which is plagued by muscle degeneration and pronounced mitochondrial lesions. Interestingly, *pink1* null flies were subsequently found to phenocopy their *parkin*-deficient counterparts and importantly, *parkin* over-expression in *pink1*^−/−^ flies were shown to be capable of rescuing all the mutant phenotypes tested, although the reverse, does not happen (Clark et al., 2006; Park et al., 2006), suggesting that Parkin acts in the same pathway but downstream of PINK1. Several follow-up studies in flies and other model systems that attempted to explain the mitochondrial phenotype focused initially on the role of Parkin/PINK1 pathway in mitochondrial dynamics, although whether the pathway promotes mitochondrial fission or fusion remains controversial to this date (for a recent review, see Burbulla et al., 2010). Besides potentially participating in mitochondrial dynamics, Parkin, as we have noted earlier, is also involved in mitochondrial biogenesis by virtue of its ability to regulate PGC-1α indirectly through its ability to down-regulate PARIS (Shin et al., 2011), a major transcriptional repressor of PGC-1α (Scarpulla, 2008). The repression of PGC-1α (a key regulator of mitochondrial biogenesis) by PARIS is expected to result in reduced mitochondrial mass, which if unregulated could compromise the ability of the cell to adapt to energy crises.

**Figure 3:**
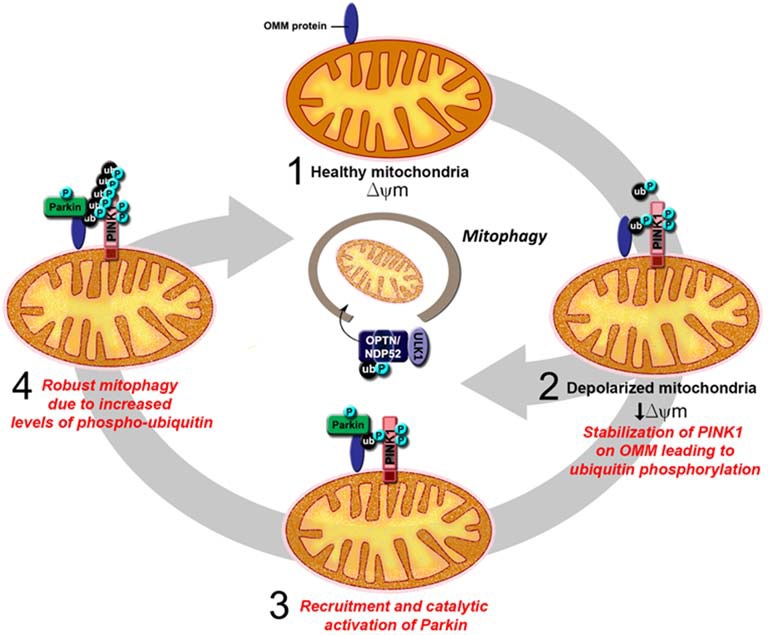
**An updated model of PINK1/Parkin-mediated mitophagy. (1)** In healthy mitochondria, there is no accumulation of PINK1 on the outer mitochondrial membrane (OMM) as the protein is rapidly imported, processed and degraded. **(2)** Upon mitochondrial depolarization, full length PINK1 accumulates on the OMM leading to the phosphorylation of ubiquitin on the surface of the mitochondria. This results in the recruitment of the autophagy receptors optineurin (OPTN) and NDP52 and the consequent activation of the mitophagy process, albeit at low level. **(3)** Parkin is also recruited to the OMM, whose latent activity becomes unmasked due to its interaction with phosphorylated ubiquitin and its phosphorylation by PINK1. **(4)** Activated Parkin promotes the polyubiqutination of mitochondrial substrates that in turn provides more ubiquitin substrates for PINK1 to phosphorylate. This amplifies the signal for the recruitment of autophagy receptors and results in robust mitophagy.

In recent years, much attention on the relationship between Parkin function and mitochondrial homeostasis has been shifted to its role in mitophagy. As several excellent reviews on Parkin/PINK1-mediated mitophagy have been written on this topic (for example, see Durcan and Fon, 2015; Pickrell and Youle, 2015), we will only briefly describe the model here, as depicted in Figure 3. Until recently, the model describes a linear sequence of events occurring in response to mitochondrial depolarization that culminates in their removal. According to the proposed model (Youle and Narendra, 2011), a key initial event that occurs upon mitochondrial depolarization is the selective accumulation of PINK1 on the outer membrane of the damaged organelle. This accumulation allows PINK1 to recruit Parkin (Okatsu et al., 2012), whose latent ubiquitin ligase activity becomes unmasked along the way in part due to its phosphorylation by PINK1 (Matsuda et al., 2010; Kondapalli et al., 2012). PINK1 also phosphorylates ubiquitin, which binds and activates Parkin (Kane et al., 2014; Koyano et al., 2014). We now know from several recent structural studies discussed above how the dual phosphorylation events mediated by PINK1 lead to the release of Parkin’s auto-inhibited state. Activated Parkin then promotes the ubiquitination and subsequent degradation of many outer mitochondrial membrane (OMM) proteins (Chan et al., 2011; Yoshii et al., 2011). During the process, Parkin-decorated mitochondria progressively cluster towards the peri-nucleus region to form “mito-aggresomes” (Lee et al., 2010), which by virtue of their association with lysosomal components are removed with time in an autophagy-dependent manner. Although this linear model explains the selectivity of the mitophagy process rather elegantly, recent discoveries revealed that a more complicated network of molecular interactions is involved in the disposal of unwanted mitochondria (Heo et al., 2015; Lazarou et al., 2015). In essence, these studies demonstrated that mitochondrial-localized PINK1 is able to recruit the autophagy receptors optineurin (OPTN) and NDP52 in the absence of Parkin and that this phenomenon alone is sufficient to trigger mitophagy, albeit at a low level. The recruitment of OPTN and NDP52 is dependent on PINK1-mediated phosphorylation of ubiquitin, which serves as autophagy signal on the mitochondria. Parkin promotes the process by amplifying the phospho-ubiquitin level generated by PINK1 that otherwise occurs at a low level due to the limited basal ubiquitin availability on mitochondria. In so doing, Parkin mediates a positive feed-forward cycle that generates more ubiquitin substrates on the OMM for PINK1 to phosphorylate that in turn facilitates the recruitment of autophagy receptors thereby enhancing the mitophagy process. Adding to this complexity is the recent elucidation by several groups that deubiquitinating enzymes (DUBs) such as USP8, USP15, USP30 and USP35 can counteract Parkin-mediated mitochondrial ubiquitination and consequently mitophagy, although they generally do not affect the recruitment of Parkin to damaged mitochondria (Bingol et al., 2014; Cornelissen et al., 2014; Durcan et al., 2014; Wang et al., 2015).

Notwithstanding the elegance of the proposed mitophagy model, whether the process occurs in post-mitotic neurons that are obligatorily dependent on mitochondrial respiration is contentious. Intuitively, one would logically accept the need for neurons to embark on mitophagy for QC reasons but perhaps in a more limited fashion. An example of this scenario is when damage of mitochondria occurs in at the distal ends of neurons. In this case, the motility of the restricted population of damaged mitochondria has been shown to be arrested. This occurs via the degradation of the mitochondrial motor adaptor protein Miro in a pathway also mediated by PINK1 and Parkin, the effect of which leading to arrested motility suggests that PINK1/Parkin pathway may quarantine damaged mitochondria prior to their clearance (Wang et al., 2011b; Ashrafi et al., 2014). Interestingly, a recent study provided evidence that Parkin-mediated neuronal mitophagy does occur *in vivo*. The investigators found that the brains of Parkin mutant flies exhibit a significantly decreased rate of mitochondrial protein turnover, which is similar to that produced by general autophagy blockade induced by the genetic ablation of *atg7*, suggesting that Parkin indeed promotes mitochondrial turnover through autophagy and that the process is physiologically relevant, albeit in the fly (Vincow et al., 2013).

Apart from mitophagy, PINK1/Parkin-dependent mitochondrial QC also occurs via an alternative pathway involving mitochondria-derived vesicles (MDVs). During various forms of mitochondrial stress, MDVs are formed from the budding of membranes from the mitochondria, which mediate the transport of damaged mitochondrial proteins and lipids to other intracellular organelles. The nature of mitochondrial stress is an important determinant of the constituents of its cargo, which in turn determine the fate of MDVs. For example, MDVs containing MAPL, an OMM-anchored protein ligase, exclude other OMM markers such as TOM20 and are selectively targeted to a subpopulation of peroxisomes (Neuspiel et al., 2008). Other MDVs that are not destined to the peroxisome fused instead with late endosomes (or multivesicular bodies), which ultimately target them for lysosomal degradation (Soubannier et al., 2012a). Importantly, the generation of late endosome-targeted mitochondria is dependent on PINK1 and Parkin and occurs under moderate mitochondrial stress as opposed to acute mitochondrial toxin treatment that leads to mitophagy. Furthermore, this phenomenon that is independent of ATG5 and LC3 does not require mitochondrial depolarization, suggesting that this pathway complements mitophagy and contributes to mitochondrial QC (Soubannier et al., 2012a,b; Sugiura et al., 2014). In another scenario, we have recently found that chronic, low dose CCCP treatment promotes Parkin-mediated mitochondrial fusion instead of mitophagy (Norris et al., 2015). In principle, active fusion could protect mitochondrial integrity by complementing damaged mitochondria with their healthy counterparts or by limiting the production of ROS. This adaptive mitochondrial fusion strategy that we have found in the presence of low but chronic mitochondrial stress is likely more physiologically relevant to the disease than high dose mitochondrial toxins commonly used to induce acute neuronal dysfunction. Importantly, we further showed that Parkin, PINK1 and α-synuclein form a regulatory circuit to regulate mitochondrial stress response, thus functionally connecting three key PD-linked genes to the pathogenesis of PD. To clarify, our result does not exclude the need for neuronal mitophagy, especially when mitochondria become chronically damage with time. It does however emphasize the need to pay close attention to the conditions used in mitophagy-related experiments and that mitochondrial QC involves not just its turnover. Whatever it is, it is evident from various studies above that Parkin appears to be involved in the entire spectrum of mitochondrial dynamics, i.e., from biogenesis, fusion/fission, intracellular movements to its exit from the cell.

## Parkin Dysregulation and PD

PD is a prevalent neurodegenerative disease that is characterized clinically by a constellation of motoric deficits arising principally from the loss of midbrain DA neurons in the substantia nigra pars compacta (SNpc) that is often accompanied by the presence of LBs in surviving neurons in the SN and other affected brain areas (Braak et al., 2003). Given the pivotal role that Parkin plays in regulating protein and mitochondrial homeostasis, and that DA neurons are usually in a heighten state of stress relative to other neuronal populations, it is perhaps not surprising to note that Parkin dysregulation can result in PD-associated DA neurodegeneration. Moreover, several studies also implicated a more direct role for Parkin in neuroprotective signaling. For example, Parkin-mediated ubiquitination was demonstrated to be important for the activation of major cellular pro-survival pathways such as the NF-κB pathway (Henn et al., 2007), or the suppression of stress-related mitogen-activated protein kinase signaling (e.g., JNK and p38; probably indirectly through its ability to counteract oxidative stress; Cha et al., 2005; Ren et al., 2009). Accordingly, loss of Parkin function would be dire for neuronal survival.

Using phosphorylated ubiquitin as a surrogate marker for mitophagy (or at least the activation of which), Fiesel et al. (2015) recently presented evidence that mitophagy is defective in the brains of PD patients. By means of phospho-specific ubiquitin immunostaining that only recognizes the presence of S65-phosphorylated ubiquitin (pS65-Ub), the authors found that the level of pS65-Ub (that is barely detectable under basal condition) is elevated in cells in response to mitochondrial stress (Fiesel et al., 2015). Importantly, they further found that pS65-Ub accumulates in human brain during aging and in post-mortem PD brain samples. Whether a (hitherto unknown) phosphatase returning pS65-Ub to its basal state is deficient in aged or sporadic PD brains that had resulted in the observed increase in pS65-Ub level is presently unclear. Nonetheless, mitophagy defects seem to be associated with PD pathogenesis. Obviously, Parkin dysfunction can also trigger impairments in neuronal protein homeostasis. Again, using PARIS as an example to illustrate this, its overexpression (via stereotactic injection of viral vector encoding PARIS) in the SN of mice was shown to result in a selective loss of DA neurons. This phenotype can be rescued by either Parkin or PGC-1α co-expression, suggesting that PARIS upregulation may underlie neurodegeneration due to Parkin activation (Shin et al., 2011). Along the same vein, Ekholm-Reed et al. (2013) recently found that the SCF substrate adapter Fbw7β is a target of Parkin during acute oxidative stress and that it accumulates in Parkin-deficient neurons. A consequence of Fbw7β accumulation is that the level of Mcl-1, an anti-apoptotic protein substrate of SCF^Fbw7β^-mediated degradation, is depleted. Loss of Parkin may thus lead to the death of DA neurons through unregulated SCF^Fbw7β^-mediated ubiquitination-dependent proteolysis of Mcl-1, which again illustrates the need to for Parkin to maintain tight homeostatic control over its substrates for optimal neuronal function. However, a more recent study demonstrated that Mcl-1 is also targeted by Parkin for degradation (Carroll et al., 2014) so the verdict regarding the relationship between Parkin and the anti-apoptotic protein Mcl-1 is currently open.

Given the neuroprotective function of Parkin, understanding how Parkin dysregulation occurs in the PD brain is an important endeavor to pursue that has obvious therapeutic implications. From the studies conducted to date, we now appreciate that Parkin dysfunction could happen under a variety of conditions. To begin with, several disease-associated mutations of Parkin directly result in the loss of its enzymatic activity. These include exon deletions that lead to the considerable truncation of the protein and missense mutations that reside on its catalytic RING2 domain. In cases where the missense mutation occurs outside of Parkin’s RING2 domain (i.e., not affecting its catalytic competency), its solubility is often altered that consequently promotes its aggregation and thereby immobilization into inclusion bodies (Ardley et al., 2003; Cookson et al., 2003; Gu et al., 2003; Muqit et al., 2004; Sriram et al., 2005; Wang et al., 2005b; Hampe et al., 2006). Interestingly, among the aggregation-producing Parkin mutations is the R275W substitution, which frequently occurs in the heterozygous state. Although the implied pathogenicity of single heterozygous Parkin mutation contradicts the widely accepted notion that Parkin mutations transmit in a classical autosomal recessive manner, one could envisage that Parkin haploinsufficiency arising from such mutations could increase the risk of heterozygous Parkin carriers for PD especially in view of the importance of optimal Parkin expression to neuroprotection. Indeed, several lines of evidence support the proposal that a single allelic hit on *Parkin* might be sufficient to cause disease (Hilker et al., 2001; Walter et al., 2004; Buhmann et al., 2005). Alternatively, some mutations of Parkin may compromise its function by destabilizing the protein and accelerating its degradation via the proteasome (Schlehe et al., 2008).

That Parkin mutations transmit in a recessive manner would suggest that the loss of Parkin function predisposes DA neurons to degeneration. An important corollary to this is that any post-translational event that promotes the loss of Parkin function could potentially mimic the effects brought about by overt mutations and be just as detrimental. Further, in view of the suggested contribution of Parkin haploinsufficiency to disease risk, it is reasonable to assume that the down-regulation of normal Parkin function mediated by such events need not result in the total abolition of its enzymatic activity to elicit a pathogenic effect. Notably, we and others have found that a wide variety of PD-linked stressors, including those that produce oxidative and nitrosative stress, induce Parkin solubility alterations and thereby its aggregation in a manner that is analogous to that brought about by several of its missense mutations (Winklhofer et al., 2003; LaVoie et al., 2005; Wang et al., 2005a). Remarkably, dopamine also modifies Parkin in a similar fashion (LaVoie et al., 2005; Wang et al., 2005a). Further, Parkin appears to be uniquely susceptible to dopamine-induced modifications compared to several other related E3 members such as HHARI, Cbl and CHIP (LaVoie et al., 2005, 2007; Wong et al., 2007). Like haploinsufficiency, the biochemical depletion of soluble Parkin levels is expected to increase the vulnerability of susceptible neurons to degeneration. Interestingly, normal Parkin in the brain also becomes progressively more detergent-insoluble with aging (Pawlyk et al., 2003), which may provide an explanation to why age represents a risk factor for PD. Besides stress-induced modifications, Parkin phosphorylation is another post-translational modification that could alter its activity. Unlike PINK1-mediated phosphorylation of Parkin that activates the enzymes, previous studies have demonstrated that Parkin phosphorylation by casein kinase 1 (CK1; at Ser-101, -127, -131, -136, -296, -378) or cyclin-dependent kinase 5 (Cdk5; at Ser-131) down-regulates its activity, and that compound phosphorylation of Parkin by both kinases further leads to its aggregation (Yamamoto et al., 2005; Avraham et al., 2007; Rubio de la Torre et al., 2009), Consistent with this, Parkin phosphorylation was found to be elevated in distinct regions of sporadic PD brains and correlates with increased levels of p25, the activator of CDK5 (Rubio de la Torre et al., 2009). Notwithstanding this, how the various phosphoserines on Parkin modulate its activity remains unclear, especially when the majority of these residues lie outside the regulatory RING0 box of the linker segment of Parkin. Similar to serine phosphorylation, tyrosine phosphorylation of Parkin at residue 143 (Y143) by the src family kinase member, c-Abl, similarly inactivates its enzyme activity and compromises its protective function both *in vitro* and *in vivo*. This form of modified Parkin is also found elevated in human post-mortem PD brains (Ko et al., 2010). Besides post-translational modifications, a number of Parkin interactors have also been demonstrated to be capable of inactivating its E3 ligase activity. These include the bcl-2-associated athanogene 5 (BAG5) and 14–3–3η whose association with Parkin reduces its activity and Nrdp1, a RING-finger containing ubiquitin ligase promotes Parkin degradation and depletes the availability of Parkin to the cell (Kalia et al., 2004; Zhong et al., 2005; Sato et al., 2006). These events if unregulated would obviously be detrimental to Parkin’s protective function.

Taken together, it is apparent that loss of Parkin function is not limited to those induced by disease-causing mutations, but also includes several biochemical and protein-protein modifications that can either alter the catalytic function of the E3 ligase directly, or indirectly through promoting its aggregation or degradation. Collectively, these mutation-independent modifications that inactivate Parkin activity provide a mechanism for Parkin dysfunction that is relevant to the pathogenesis of sporadic PD. At the same time, they also suggest therapeutic directions in managing the disease. Interesting, whereas *S*-nitrosylation of Parkin leads to its inactivation, sulfhydration of Parkin has recently been shown to enhance its activity (Vandiver et al., 2013). Moreover, the levels of sulfhydrated Parkin is markedly depleted in PD brains while nitrosylation increased in tandem, suggesting that hydrogen sulfide donors may counteract S-nitrosylation-mediated inactivation of the enzyme and may accordingly be therapeutic. Similarly, the c-Abl inhibitor, nilotinib, is also protective against DA neuronal loss in a toxin-induced model of PD, although curiously the level of phospho-Y143 Parkin appears to be unchanged (Karuppagounder et al., 2014). These recent studies suitably illustrate the importance of mechanistic insights in guiding therapeutics development.

## Parkin Dysregulation and Other Neurodegenerative Diseases

Besides PD, accumulating evidence suggests that Parkin dysfunction may also contribute to the pathogenesis of other neurodegenerative diseases including AD and ALS. Pathologically, the AD brain is characterized by the presence of extracellular amyloid aggregates and intra-neuronal hyperphosphorylated tau aggregates known as neurofibrillary tangles (NFTs). Among the first hints suggesting a relationship between Parkin and AD is the finding by Nemes et al. (2004) that Parkin is a major component cross-linked by gamma-glutamyl-epsilon-lysine bonds in AD-associated NFT. More recently, Corsetti et al. (2015) showed that an N-terminal truncated tau species found in AD individuals can induce deregulated mitophagy by aberrant recruitment of Parkin that result in excessive mitochondrial turnover, synaptic deterioration and neuronal death, thus providing a Parkin-related mechanism in the pathogenesis of AD. Notably, a number of earlier studies have already documented abnormal tau hyperphosphorylation in patients with Parkin mutations (van de Warrenburg et al., 2001; Sánchez et al., 2002), suggesting that Parkin deficiency may precipitate tau pathology. Consistent with this, the accumulation of tau was observed during ageing in Parkin null mice (Rodríguez-Navarro et al., 2007). Interestingly, overexpression of mutant tau in mice against Parkin null background promotes cerebral and systemic amyloidosis (Rodríguez-Navarro et al., 2008) that is accompanied by impairments in memory and exploratory behaviors (Navarro et al., 2008), suggesting that Parkin may also affect amyloidogenesis. Indeed, Rosen et al. (2006) reported that Parkin could lower the level of amyloid burden and protect against its toxicity (albeit in muscle cells). In a series of follow up studies, the same group and others demonstrated the ability of Parkin to facilitate the clearance of the pathogeneic amyloid β42 by reversing its negative effects on the proteasome and also by enhancing beclin-dependent autophagy (Burns et al., 2009; Rosen et al., 2010; Khandelwal et al., 2011). Not surprisingly, Parkin overexpression in an AD mouse model (that is based on altered amyloid precursor protein processing, i.e., APPswe/PSEN1ΔE9 mutant) was found to reduce β-amyloid load and improve hippocampal long term potentiation (Hong et al., 2014). Similar outcomes were also observed when AD animals were treated with nilotinib, which increases parkin solubility and its interaction with Beclin one leading to enhanced amyloid clearance and cognitive performance (Lonskaya et al., 2013).

In a parallel development, several groups working on FUS/TLS and TDP-43 related ALS found an association between these ALS-mutants and Parkin. In an elegant functional genomics study, Yeo’s group found that among the 45 RNA species that were reduced following depletion of TDP-43 or FUS/TLS, one of these encodes Parkin (Lagier-Tourenne et al., 2012). Consistent with this, mice carrying a knock-in copy of mutant TDP-43 exhibit reduced level of Parkin expression (Stribl et al., 2014). Thus, Parkin deficiency may also be interwoven with the pathogenic events in ALS. Indeed, Hebron et al. (2014) recently demonstrated the ability of Parkin to reverse TDP-43-mediated cell death. Apparently, mitochondrial dysfunction may be an underlying denominator responsible for neuronal death in ALS as well. Notably, altered mitochondrial QC was observed in the TDP-43 knock in mutant mice (Stribl et al., 2014). Moreover, mutations in the autophagy/mitophagy receptor OPTN are associated with ALS (Maruyama et al., 2010) and mutant OPTN are apparently incapable of facilitating Parkin/PINK-mediated mitophagy (Wong and Holzbaur, 2014). Finally, despite the anatomical and functional relatedness of HD and PD (i.e., both affect the basal ganglia circuitry and result in movement disorders), only a handful of reports suggest that Parkin deficiency may be a contributor to striatal neurodegeneration in HD. In an earlier study, Parkin was found to protect against the toxicity induced by expanded polyglutamine proteins (Tsai et al., 2003). Subsequent to this, another group found that partial suppression of Parkin aggravates the phenotype in the huntingtin R6/1 mutant mice, albeit only slightly (Rubio et al., 2009). More recently, Parkin/PINK-mediated mitophagy was shown to promote neuroprotection in fly and mouse models of HD (Khalil et al., 2015).

In sum, it is evident from the above discussion that Parkin deficiency is relevant not just to PD but may also be relevant to several other major neurodegenerative diseases. Obviously, this begs the question on why mutations in Parkin are uniquely causative of PD. Although we remain unclear about this, one possible explanation is that human DA neurons are selectively vulnerable to the loss of Parkin function perhaps by virtue of their heighten stress, i.e., Parkin deficiency in this case is the principal driver of disease pathogenesis. In AD and ALS, the depletion of Parkin occurs against the backdrop of other pathogenic factors; the combination of which is likely to aggravate neuronal loss, i.e., Parkin dysfunction is the co-driver. Arguably, some may consider Parkin’s role in these diseases as a passenger, i.e., a consequence of AD or ALS-related pathogenic factors going awry, although several reports demonstrated that Parkin overexpression is beneficial in these cases. Whether Parkin is acting as a driver, co-driver or passenger, we probably could all accept that its optimal expression is important for neuronal homeostasis.

## Concluding Remarks

It has been 15 years or so since the function of Parkin was originally elucidated. Certainly, we now know much more about the various aspects of the E3 than we did before. Yet, it remains rather amazing at the same time to note that a single ubiquitin ligase could possess such multi-functionality that enables it to perform diverse types of ubiquitination (including mono, K48- and K63-linked ubiquitin modifications) to support a plethora of cellular processes, as well as lend itself as a broad spectrum neuroprotectant. As articulated above, emerging evidence also suggests its relevance to several major neurodegenerative diseases besides PD, and interestingly, to cancer development as well. Thus, Parkin appears to be one of a kind. Accordingly, its activity regulation must be exquisitely controlled in different cell types under different conditions for optimal cellular function. On a related note, given the amazing properties of this RING-containing E3, we thought that it would be appropriate to end this article by suggesting that Parkin be (affectionately) nicknamed as the “Lord of the RING”. Obviously, we are bias here and concede that some readers may consider Parkin as just a “RING-bearer”. Be it the lord or a RING-bearer, we cannot deny the fact that Parkin does have some very enchanting properties.

## Author Contributions

CZ and LH drafted the manuscript. CZ generated the table. LH illustrated the figures. TPY provided intellectual inputs and critiques, and contributed to text. KLL conceptualized the flow of the article, revised the draft, polished the text and finalized the manuscript.

## Conflict of Interest Statement

The authors declare that the research was conducted in the absence of any commercial or financial relationships that could be construed as a potential conflict of interest.
